# Effects of a Probiotic Formulation on Seasonal Allergic Rhinitis in Adults—A Randomized Double-Blind Placebo-Controlled Trial: The Probiotics for Hay Fever Trial

**DOI:** 10.3389/fnut.2022.887978

**Published:** 2022-05-23

**Authors:** Karin Ried, Nikolaj Travica, Yeah Paye, Avni Sali

**Affiliations:** ^1^National Institute of Integrative Medicine (NIIM), Melbourne, VIC, Australia; ^2^Torrens University, Adelaide, SA, Australia; ^3^Discipline of General Practice, The University of Adelaide, Adelaide, SA, Australia; ^4^Food and Mood Centre, Deakin University, Geelong, VIC, Australia

**Keywords:** hay fever, seasonal allergic rhinitis, probiotics, symptoms, quality of life, T-helper cell ratio

## Abstract

**Background:**

Seasonal-allergic-rhinitis (hay fever) affects approximately 4.6 million (20%) Australians each year. Hay fever manifests as runny/blocked nose and often itchy/sore/swollen eyes, with symptoms greatly impacting the quality of life. Rescue medications such as antihistamines are often needed to restore function, but they may trigger some other unwanted side effects. Probiotics have shown promise to reduce hay fever symptoms.

**Objective:**

In this randomized double-blind placebo-controlled 12-week trial, we aimed to assess the tolerability and efficacy of the probiotic formula “NC-Seasonal-Biotic” on symptoms, quality-of-life, and immunological and microbial factors.

**Methods:**

Adults, who had previously suffered from hay fever symptoms, were screened for eligibility and randomly allocated to probiotic or placebo trial powder. Treatment effectiveness was assessed by questionnaires, daily total-nasal-symptom-score, and weekly rhinoconjunctivitis quality-of-life questionnaire. Secondary outcome measures included immunological parameters such as T-cell immunity (Th1/Th2 ratio) and the stool-microbiome analysis. Tolerability was assessed weekly by the gastrointestinal symptom scale.

**Results:**

Recruitment and follow-up were challenging around the 2020/2021 hay fever season in Melbourne, Australia, due to the harsh COVID-19 restrictions and extended lockdowns. Out of the 82 adults enrolled in this study, 75% participated (*n* = 60), and half (*n* = 40) completed the 10–12-week intervention period. In the intention-to-treat analysis, no significant differences in hay fever symptoms were apparent between the groups, while quality-of-life trended toward greater improvement in the active group. Intention-to-treat analysis was confounded due to a third of all participants not completing the full 10–12-week-intervention period. Subgroup analyses of the participants (*n* = 40) completing the full 10–12-week study period revealed a significantly greater reduction in symptoms in the active group compared with the placebo group, including runny nose (*p* = 0.04) and itchy eyes (*p* = 0.01). Furthermore, the active group reported significant improvements in the quality-of-life, including more functionality during the day (*p* = 0.05), better sleep (*p* = 0.005), less fatigue (*p* = 0.04), less thirst (*p* = 0.007), and less irritability (*p* = 0.007). Immunological parameters, measured by T-helper cell ratio (Th1/Th2), improved significantly in the active group compared with the placebo group. Most microbial changes were not statistically different between the groups. The trial powder was generally well tolerated.

**Conclusion:**

Our study suggests the probiotic formula “NC-Seasonal-Biotic,” taken for 10–12 weeks, as effective in reducing hay fever symptoms, such as runny nose and itchy eyes, and improved the quality-of-life and immunological parameters while being well tolerated.

**Clinical Trial Registration:**

[www.ClinicalTrials.gov], identifier [ACTRN126200 01078943].

## Introduction

Seasonal allergic rhinitis, or hay fever, affects approximately 20% of the Australian population, or approximately 4.6 million people, according to the 2017/2018 Australian National Health Survey (1).

A recent systematic review and meta-analysis of 22 trials involving more than 800 participants and investigating the effectiveness of probiotics on symptoms of seasonal allergic rhinitis concluded that probiotics significantly reduced nasal symptoms during peak hay fever season and significantly improved the quality of life and immunological parameters such as T-helper cell response (2).

In addition, a more recent study by Dennis-Wall et al. (3), involving 161 participants and investigating a three-strain probiotic for 8 weeks, found that the symptoms and quality of life improved significantly in the probiotic group compared with the placebo group.

Standard assessment tools for nasal and ocular symptoms included the Total Nasal Symptom Score (TNSS) (4) and the Rhinoconjunctivitis Symptoms Score (5, 6).

The Mini Rhinoconjunctivitis Quality of Life Questionnaire (Mini RQLQ) (7) assessed the impact and severity on the quality of life of sufferers with hay fever and provided a useful primary screening tool for the selection of subjects in the studies.

Probiotics are suggested to stimulate gut-associated immunity through cytokine expression and T-helper (Th) cell response (8), whereby Th1 cells are associated with pro-inflammatory cytokines and Th2-cells with anti-inflammatory cytokines. Changes in immunological parameters, such as the Th1:Th2 ratio, can provide useful insights into the effectiveness of probiotics on seasonal allergies/hay fever.

For example, a significantly lower Th1:Th2 ratio in the probiotic group was associated with an improvement in hay fever symptoms in a meta-analysis of five studies (2, 9–13).

Microbial composition in the gut also plays a role in susceptibility to allergy. A large gut study involving more than 1,800 American adults with allergies, including seasonal pollen allergies, found low microbial diversity, in particular, reduced *Clostridiales* and increased *Bacteroidales* species (14).

In this randomized double-blind placebo-controlled 12-week trial, we assessed the tolerability and efficacy of the Nutrition-Care Probiotic Formula “Seasonal Biotic” on symptoms, quality of life, and immunological and microbial factors in adult sufferers with hay fever.

## Methods

### Trial Design and Participants

The randomized double-blind placebo-controlled trial of 12-week duration was conducted in the 2020/2021 Hay fever Season between October 2020 and January 2021 at the National Institute of Integrative Medicine (NIIM) in Melbourne, Australia. Participants were recruited through the NIIM website, newsletter, flyers, and social media in Melbourne.

This study was approved by the NHMRC endorsed NIIM Human Research Ethics Committee and acknowledged under Clinical Trial Notification by the Australian Therapeutic Goods Administration (TGA). Participating patients provided written informed consent. This study is registered on the Australian New Zealand Clinical Trial Registry ACTRN12620001078943.

### Screening and Inclusion Criteria

Adults 18-75 years old with self-reported hay fever/seasonal allergic rhinitis were screened for eligibility by the modified Mini RQLQ (7).

We excluded those with allergic rhinitis due to other causes than seasonal hay fever, such as allergy to house dust mites or animal hair. Adults diagnosed with respiratory disease, e.g., COPD, asthma, mast cell activation syndrome, on medication for respiratory illness, taking antidepressants, immunotherapy or immunosuppressive medications, antibiotics, or intolerance to probiotics, fructooligosaccharides, or sorbitol, were also excluded.

### Randomization, Allocation, and Blinding

Consenting eligible participants were randomly allocated to either the active probiotic or placebo group using a computer-generated permuted random number table provided by an independent researcher not involved in recruitment and data collection.

Active and placebo powder was packaged offsite in identical sachets. Participants, as well as investigators and research assistants, were blinded to the group allocation. The blinding success of patients was evaluated by questionnaires at the end of the trial.

### Trial Medication and Procedure

The trial supplement sachets contained either the active probiotic or a placebo powder and were identical in appearance and mass (1.5 g). For the probiotic powder, each 1.5 g sachet contained 10 billion CFU total probiotics, including *Lactobacillus reuteri GL 104, Lactobacillus plantarum LPL28, Lactobacillus rhamnosus MP108*, and *Bifidobacterium lactis BI04*, and fructooligosaccharide (prebiotic), Nutriose (prebiotic), and 840 mg Sorbitol (Table 1: active powder). The placebo powder consisted of fructooligosaccharide, Nutriose, and 840 mg Sorbitol.

**TABLE 1:** Probiotic active powder composition.

Active group			

SEASONAL BIOTIC (10^10^CFU/1.5 g)	Billion CFU/per sachet (1,500 mg)	Composition per sachet (mg/1,500 mg)	%w/w
Bifidobacterium lactis BI04	2.7	16.2	1.08
Lactobacillus plantarum LPL28	5.595	55.95	3.73
Lactobacillus reuteri GL-104	0.405	12	0.8
Lactobacillus rhamnosus MP-108	1.305	39	2.6
Flavor		26.25	1.75
sorbitol		975	65
dextrin		210	14
FOS (fructooligosacharides)		165.6	11.04
Total input		1,500	100

Participants were instructed to take one 1.5 g trial powder sachet daily over 12 weeks with water or food. The trial supplement was manufactured and supplied by Nutrition Care/Ausnutria.

Participants were asked to avoid any other probiotics during the study.

### Study Timeline

The 12-week intervention period was to cover a 4-week pre-peak period (October 2020) and an 8-week peak period (November/December 2020), with final follow-up measures taken in January 2021.

**FIGURE 1:**
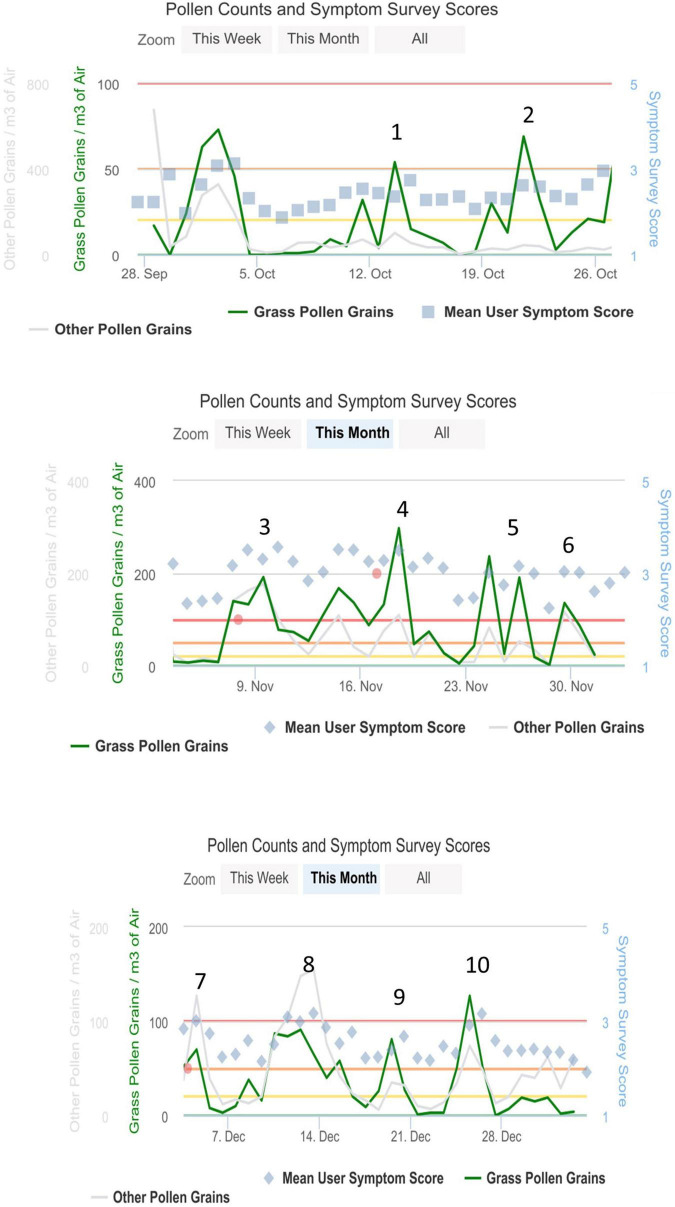
Pollen peaks in hay fever season from October to December 2020 in Melbourne, VIC, Australia, Ref: Melbourne Pollen Count (19).

The daily pollen count in Melbourne was recorded through the Melbourne Pollen App, and data were incorporated into the analysis, whereby weekly symptom scores were averaged around pollen peaks (Figure 1). Pollen peak 1 was at the beginning of October 2020, and the end study pollen peak 10 was observed at the end of December 2020.

### Assessments

#### Primary Outcome Measures

##### Quality of Life Questionnaire

The Mini RQLQ (7) was used as a screening tool for eligibility and as a primary outcome assessment tool during the trial.

The modified Mini RQLQ consisted of 18-items on a 7-point Likert scale ranging from 0 = not troubled to 6 = extremely troubled (Supplementary Appendix 1).

At screening, participants had to score at least 16 points (i.e., > 5 symptoms × score of “3 = moderately troubled” to be eligible for the trial).

During the trial, participants were instructed and reminded by SMS to record their impact of hay fever symptoms on activities weekly in an online questionnaire every weekend during the trial. Responses during the first pollen peak at the start of the study were used as the baseline and compared with responses at the last pollen peak at the end of the trial.

##### Total Nasal and Eye Symptoms

Total nasal and ocular (eye) symptom scores were assessed daily by the modified TNESS-Q 8-item (3 items relating to nasal symptoms and 5 items to eye symptoms) 7-point Likert scale ranging from “0 = no symptoms” to “6 = severe symptoms/extremely troublesome” (Supplementary Appendix 2).

Participants were instructed to record symptoms daily on an online questionnaire, also assessing any intake of rescue medication. For analysis, a 7-day average was calculated around pollen peak times. The baseline peak was the first pollen peak after enrollment, and the end peak was the last pollen peak in the hay fever season. While the baseline peak varied dependent on when participants enrolled, the end pollen peak was fixed for all participants (25 December 2021).

Symptom scores were adjusted if participants had taken rescue medications.

#### Secondary Outcomes

##### Tolerability

Tolerability of the trial powder was assessed by the Gastrointestinal Symptom Rating Scale (15), a 13-item 7-point Likert scale ranging from 0 = no discomfort to 6 = very severe discomfort (Supplementary Appendix 3).

Participants were instructed and reminded by weekly SMS to record their gastrointestinal symptoms in an online questionnaire every weekend during the trial.

##### Immunological Parameters and Inflammatory Markers

Immunological parameters in the form of T-helper cell ratios (Th1/Th2) were assessed by blood test and the inflammatory marker/cytokine test panel by Nutripath Pathology, Melbourne, Australia (16).

The test panel consisted of pro-inflammatory cytokines associated with Th1 induction, including interleukin-1 (IL1), IL6, IL7, IL8, IL17, TNF-α, and TNF-β, and anti-inflammatory cytokines associated with Th2 induction, specifically GM-CSF, IL2, IF4, IL5, IL10, IL12, IL13, INF-γ, and TGF-β.

Cytokine levels were assessed using a blood test during the pollen peak at the end of the study, and Th1/Th2 ratios were estimated by calculating each pro-inflammatory cytokine to anti-inflammatory cytokine ratio. Lower Th1/Th2 ratios are associated with a lower inflammatory profile.

##### Stool Test/Gut Microbiome

Participants were provided with a commercially available complete microbiome test kit from Nutripath Pathology, Melbourne, Australia (17) at baseline and at 12 weeks and were instructed to collect a stool sample within a few days of their baseline and end of study appointment. The microbiome test consisted of a comprehensive profile of commensal bacterial species in colony-forming units (CFU)/g stool by multiplex qPCR-DNA analysis undertaken by an accredited pathology laboratory. Commensal bacterial species included *Bacteroidetes*, *Firmicutes*, opportunistic bacteria, and normal gut flora. The report was used to assess microbial richness, diversity, and *Firmicutes/Bacteroidetes* ratio.

The stool test also assessed the presence of *Helicobacter pylori.* In the case of a positive test result, participants were advised to organize an *H. pylori* breath test and antibiotic treatment through their GP. Participants were subsequently excluded from this study, as antibiotic treatment would have interfered with the study intervention of probiotic intake.

### Blinding

Blinding success was assessed at the completion of the trial, whereby participants were asked whether they thought to have been on the active or placebo, or whether they were unsure.

### Sample Size

A sample size of 80 participants (*n* = 40 in each group) was calculated based on the following assumptions:

(a) To detect a difference of 4-point score (12 points to 8 points (*SD* = 6) on the modified TNESS Symptoms score (48 point max, 8 question Likert scale 0–6) between the active treatment (*n* = 40) and control (*n* = 40) with 80% power and 95% confidence;

(b) To account for 10% drop-out or non-attendance at all appointments.

### Analysis

Descriptive statistics and comparative analyses were performed using SPSS (PASW version 26). Statistical significance was set at *p* < 0.05.

Total scores from 7-point Likert scale variables were treated as continuous variables, differences between groups were analyzed using the Student’s *t*-test, and categorical variables were analyzed using the chi-square test.

Intention-to-treat analysis was performed with available data points for all outcome measures. In addition, we undertook subgroup analyses with participants who had completed the full 10–12-week intervention for primary outcome measures, including the total symptom scores and quality of life.

We calculated 7-day average symptom scores around pollen peaks and adjusted symptoms scores by medication intake, if applicable.

The baseline peak was the first pollen peak after enrollment, and the end peak was the last pollen peak in the hay fever season. While the baseline peak varied dependent on when participants enrolled, the end pollen peak was fixed for all participants (25 December 2021).

## Results

### Recruitment/Withdrawals/Completion

Out of the 82 enrolled in this study, a total of 26 (32%) did not continue to participate. The majority of those (*n* = 18, 21%) withdrew early in the study having changed their mind about participating, 4 were excluded due to testing positive for Helicobacter pylori infection (5%), and four in the active group (4 out of 41, 10%) due to gastrointestinal intolerability of the trial powder.

A total of *n* = 60 participants completed the trial, with about twice as many females than males. The average age was 50 years, and the BMI average was 25.6 kg/m^2^. Approximately half of the participants had a BMI in the normal range, 37% were overweight, and 14% were obese. There were no significant differences in baseline characteristics between the groups (Table 2).

**TABLE 2:** Demographics/baseline characteristics.

Demographics	All (*n* = 60)		Active (*n* = 31)	Placebo (*n* = 29)	*p*-value
	Mean (*SD*)	Range	Mean (*SD*)	Mean (*SD*)	
Age (years)	50.1 (13.0)	18–75	51.9 (14.3)	48.1 (11.3)	ns
BMI (kg/m^2^)	25.6 (5.1)	18–43	26.1 (5.0)	25.2 (5.2)	ns
**BMI categories**	***N* (%)**		***N* (%)**	***N* (%)**	
Normal (18– < 25 kg/m^2^) Overweight (25– < 30 kg/m^2^) Obese (≥ 30 kg/m^2^)	30 (49) 22 (37) 8 (14)		13 (41) 15 (47) 11 (3)	16 (54) 9 (31) 4 (15)	ns
Male/female	22/38 (37/63)		13/18 (42/58)	9/20 (31/69)	ns

*BMI, body mass index; kg/m^2^, kilogram/meter-squared; N, number; ns, not significant; SD, standard deviation.*

**FIGURE 2:**
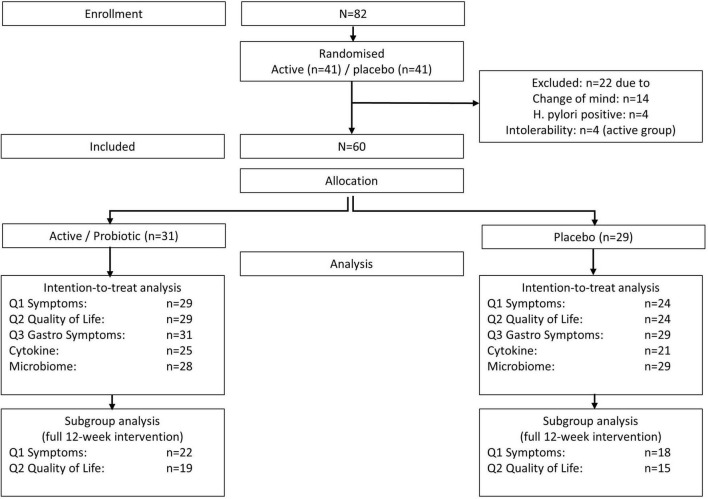
Flowchart.

Not all of the 60 participants provided comparative data (end of study and baseline) for all outcome variables, ranging from 72% for cytokine analysis to 100% for tolerability (gastrointestinal symptoms), with primary outcome measures of symptoms (Q1) and quality of life (Q2) being available for *n* = 53 (88%) of participants (Table 3 and Figure 2).

**TABLE 3:** Participants and available data.

Outcome measure	N total (active/placebo)	% of completed (*N* = 60)
Q1 Daily symptom questionnaire	53 (29/24)	88%
Q2 Weekly quality of life questionnaire	53 (29/24)	88%
Q3 Weekly gastrointestinal symptom Q	60 (31/29)	100%
Cytokine analysis	43 (25/18)	72%
Stool microbiome analysis	57 (28/29)	95%

*N, number; Q, questionnaire.*

As recruitment was ongoing throughout the hay fever period due to hesitant enrollment at the beginning of the period (October 2020), not all enrolled participants received a 12-week intervention before the end of the study (the last pollen peak was in December 2020). Therefore, we conducted a subgroup analysis with the participant who took the 12-week intervention (40 out of 53, 75%) for meaningful analysis of primary outcome measures, symptoms, and quality of life.

### Total Nasal and Eye Symptom Questionnaire

We conducted an intention-to-treat-analysis with all available data (*n* = 53), comparing the end of study symptoms at pollen peak 10 (25 December 2021) to baseline at peak 2 (20 October 2021). As 25% (13 out of 53) of the participants started later than peak 2, we adjusted their baseline using their available data at the following peaks for intention-to-treat analysis. Six of the 13 participants’ baseline was taken at peak 3 (9 November 2021, 2 weeks later), five participants’ baseline was taken at peak 4 (20 November 2021, 4 weeks later), and two participants started at peak 6 (30 November 2021, 6 weeks later) (Figure 1).

Due to the limited intervention period for 25% of the participants likely influencing results, we also undertook a subgroup analysis with *n* = 40 participants who had started the intervention at the beginning of the hay fever season in early October 2021.

Nasal and ocular symptoms tended to improve in both groups, albeit no significant differences were observed in the intention-to-treat analysis.

While 42% (22 out of 53) of participants stated to have taken rescue medication during pollen peaks, only 28% (15 out of 53) had taken these for 4 or more out of 7 days. The most common type of rescue medication was oral antihistamines (*n*_active/placebo_ = 8/9), while a small number of participants took oral or nasal steroids (*n*_a/p_ = 3/2) at baseline. Rescue medication intake was comparable in both groups at baseline and 12 weeks, with a small number of participants having decreased medication intake over time (*n*_a/p_ = 4/6) (Table 4a).

**TABLE 4:** Total nasal and eye symptom score and rescue medication intake.

	Variable	Group	*N*	Baseline	12 weeks	Within group	Active vs. placebo between groups	
					
			Total *N* = 53	Mean ± *SD*	Mean ± *SD*	Mean change ± *SD*	Mean diff ± *SE*	*p*-value

(a) All (*n* = 53)	Nasal symptoms							
	**Stuffed nose**	**Active**	29	15.9 ± 10.8	9.6 ± 8.6	−6.2 ± 10.3	0.5 ± 3.0	ns
		**Placebo**	24	16.6 ± 9.6	9.9 ± 10.5	−6.7 ± 11.7		
	**Itchiness sneezing**	**Active**	29	14.9 ± 8.4	9.7 ± 7.4	−5.2 ± 10.3	0.7 ± 3.0	ns
		**Placebo**	24	15.6 ± 9.5	9.8 ± 9.8	−5.8 ± 11.7		
	**Runny nose**	**Active**	29	14.1 ± 10.0	8.1 ± 8.9	−6.2 ± 10.3	−0.9 ± 3.3	ns
		**Placebo**	24	14.0 ± 9.3	8.8 ± 10.9	−5.2 ± 11.8		
	**Ocular symptoms**							
	**Itchy eyes**	**Active**	29	12.4 ± 10.2	6.6 ± 8.5	−5.8 ± 11.1	−1.7 ± 3.1	ns
		**Placebo**	24	12.6 ± 10.3	8.5 ± 8.9	−4.1 ± 11.4		
	**Gritty eyes**	**Active**	29	9.1 ± 11.4	6.2 ± 9.1	−3.0 ± 10.7	0.4 ± 2.6	ns
		**Placebo**	24	8.8 ± 8.7	5.4 ± 7.8	−3.4 ± 8.5		
	**Red eye**	**Active**	29	7.0 ± 10.2	4.3 ± 7.1	−2.6 ± 9.0	0.5 ± 2.4	ns
		**Placebo**	24	9.0 ± 8.4	5.9 ± 8.7	−3.1 ± 8.5		
	**Watery eyes**	**Active**	29	8.8 ± 9.8	5.6 ± 7.9	–3.2 ± 10.0	−0.4 ± 2.6	ns
		**Placebo**	24	9.1 ± 9.2	6.0 ± 8.4	−3.2 ± 8.7		
	**Puffy eyes**	**Active**	29	6.7 ± 9.3	5.3 ± 8.1	−1.3 ± 10.1	−1.0 ± 2.6	ns
		**Placebo**	24	8.5 ± 9.0	6.0 ± 8.4	−2.4 ± 8.5		
	**TNESS 8 items**	**Active**	29	88.9 ± 67.8	55.4 ± 55.5	−33.4 ± 74.8	0.4 ± 20.5	ns
		**Placebo**	24	94.3 ± 65.8	60.4 ± 66.6	−33.9 ± 73.5		
	**TNSS 3 items**	**Active**	29	44.9 ± 25.9	27.4 ± 21.6	–17.4 ± 31.0	0.3 ± 8.9	ns
		**Placebo**	24	46.2 ± 26.6	28.5 ± 30.5	−17.7 ± 33.3		
	**TESS 5 items**	**Active**	29	44.0 ± 45.7	28.0 ± 36.0	−16.0 ± 47.4	0.2 ± 12.6	ns
		**Placebo**	24	48.0 ± 43.8	31.8 ± 40.1	−16.2 ± 43.5		

			***N***	**Baseline days/week**	**12 weeks days/week**	**Within group days/week**	**Within group med change** ^##^	
						
**All (*n* = 53)**	**Rescue medication** ^#^		**Yes/no**	**Mean** ± ***SD***	**Mean** ± ***SD***	**Mean change** ± ***SD***	***N***	

	**1–7 days/week**	**Active**	11/18	2.1 ± 3.0	1.3 ± 2.6	−0.6 ± 1.7	Decrease: 4 no change: 25	
		**Placebo**	11/13	1.9 ± 2.5	0.8 ± 1.7	−0.95 ± 2.4	Decrease: 6 no change: 17 Increase: 1	ns
	** ≥ 4 days/week**	**Active**	8/21			−4.3 ± 2.5	As above	
		**Placebo**	7/17			−4.5 ± 1.8	As above	ns

**(b) Subgroup (*n* = 40)**	**Nasal symptoms**		***N***	**Mean** ± ***SD***	**Mean** ± ***SD***	**Mean change** ± ***SD***	**Mean diff** ± ***SE***	***p*-value**

**10–12 week intervention**	**Stuffed nose**	**Active**	22	15.4 ± 10.0	10.4 ± 8.9	−5.0 ± 7.3	−1.8 ± 2.6	ns
		**Placebo**	18	14.5 ± 9.6	11.4 ± 11.0	−3.1 ± 9.0		
	**Itchiness sneezing**	**Active**	22	13.8 ± 6.6	9.5 ± 7.4	−4.3 ± 6.8	1.3 ± 2.3	ns
		**Placebo**	18	14.3 ± 8.1	11.2 ± 10.3	−3.1 ± 7.5		
	**Runny nose**	**Active**	22	14.3 ± 8.4	8.5 ± 8.9	−5.9 ± 8.8	−5.1 ± 2.5	0.04
		**Placebo**	18	11.1 ± 7.8	10.3 ± 11.5	−0.7 ± 6.7		
	**Ocular symptoms**							
	**Itchy eyes**	**Active**	22	12.6 ± 9.0	6.3 ± 8.1	−6.3 ± 8.0	−6.1 ± 2.4	0.01
		**Placebo**	18	10.3 ± 9.4	10.1 ± 9.0	−0.2 ± 7.0		
	**Gritty eyes**	**Active**	22	9.2 ± 10.4	6.3 ± 9.1	−2.9 ± 7.1	−2.0 ± 2.0	ns
		**Placebo**	18	6.9 ± 8 0	6.0 ± 7.6	−0.9 ± 5.1		
	**Red eyes**	**Active**	22	6.3 ± 8.6	4.2 ± 6.8	−2.0 ± 4.7	−1.2 ± 1.6	ns
		**Placebo**	18	7.5 ± 8.2	6.6 ± 8.7	−0.9 ± 5.5		
	**Watery eyes**	**Active**	22	9.0 ± 8.1	5.6 ± 7.6	−3.3 ± 5.4	−2.1 ± 1.6	ns
		**Placebo**	18	7.9 ± 8.9	6.7 ± 8.4	−0.9 ± 5.1		
	**Puffy eyes**	**Active**	22	6.2 ± 7.1	5.1 ± 7.9	−1.1 ± 4.7	−1.1 ± 1.6	ns
		**Placebo**	18	7.0 ± 8.6	7.0 ± 8.9	0 ± 5.1		
	**TNESS 8 items**	**Active**	22	86.6 ± 52.3	55.8 ± 53.2	−30.8 ± 37.1	−20.8 ± 12.4	ns
		**Placebo**	18	79.4 ± 61.4	69.4 ± 67.2	−10.1 ± 41.5		
	**TNSS 3 items**	**Active**	22	43.5 ± 20.9	28.3 ± 21.5	−15.1 ± 19.5	−8.2 ± 6.5	ns
		**Placebo**	18	39.8 ± 24.4	32.9 ± 32.0	−6.9 ± 21.3		
	**TESS 5 items**	**Active**	22	43.2 ± 36.2	27.5 ± 34.3	−15.7 ± 23.3	−12.5 ± 7.5	0.07
		**Placebo**	18	39.6 ± 41.0	36.4 ± 40.1	−3.2 ± 24.2		

			***N***	**Baseline days/week**	**12 weeks days/week**	**Within group days/week**	**Within group med change** ^##^	
						
**Subgroup (*N* = 40)**	**Rescue medication** ^#^		**Yes/no**	**Mean** ± ***SD***	**Mean** ± ***SD***	**Mean change** ± ***SD***	***N***	

	**1–7 days/week**	**Active**	9/13	2.8 ± 3.0	1.4 ± 2.7	−0.5 ± 1.3	Decrease: 3 no change: 19	
		**Placebo**	8/10	2.0 ± 2.8	0.9 ± 1.8	−1.1 ± 2.6	Decrease: 5 no change: 12 Increase: 1	ns
	** ≥ 4 days/week**	**Active**	6/16	6.7 ± 0.8	5.0 ± 2.9	−1.7 ± 2.3	As above	
		**Placebo**	6/12	5.7 ± 1.2	1.8 ± 2.6	−3.8 ± 2.5	As above	ns

*(a) All available data with baseline peak 2 (20 October 2020) and end of study peak 10 (25 December 2020) adjusted for intention-to-treat analysis.*

*(b) Subgroup of participants who received full 10–12 week intervention.*

*N, number; SD; standard deviation; ns, not significant; med, medication; TNESS, total nasal eye symptom score (8 items); TNSS, total nasal symptom score (3 items); TESS, total eye symptom score (5 items), ^#^Rescue medication includes oral antihistamine and nasal and oral steroids; ^##^medication change includes type and frequency of medication, e.g., antihistamines 4 days/week.*

**FIGURE 3:**
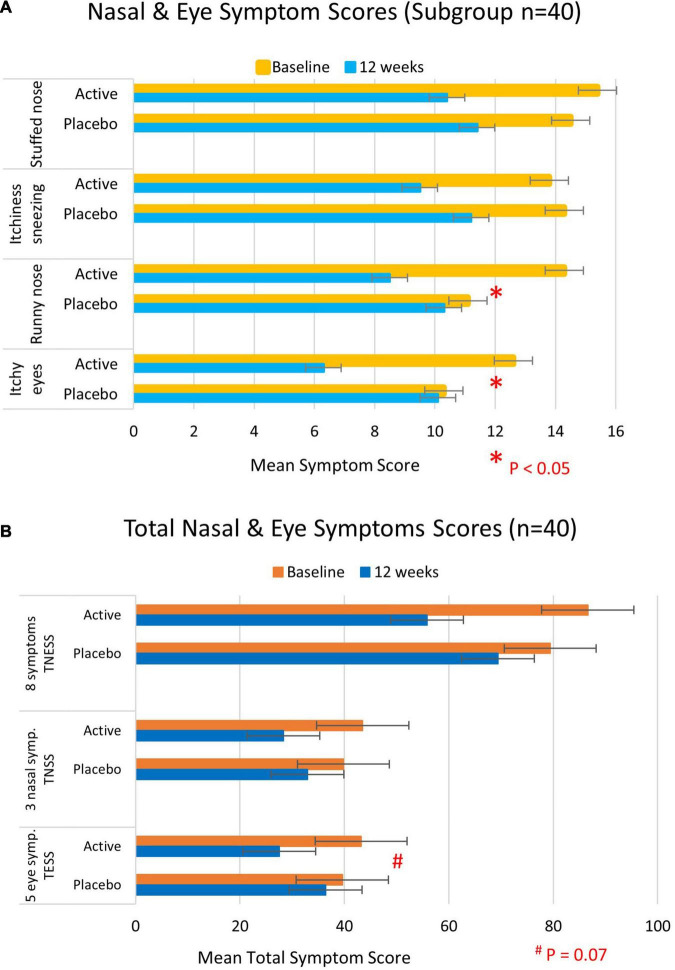
Total nasal and eye symptom scores at baseline (yellow/orange bars) and 12 weeks (blue bars) of participants who took the full course of 10–12 week trial intervention (*n* = 40). **(A)** Individual symptom scores and **(B)** total nasal and/or eye symptom scores. Higher scores represent more severe symptoms.

In contrast to the intention-to-treat analysis, significant differences in symptom scores were apparent in the subgroup analysis of 40 participants who had taken the intervention for a 10–12 week period. These included a significant improvement in the runny nose (mean diff ± SE: −5.1 ± 2.5, *p* = 0.04) and itchy eye symptoms (mean diff ± SE: −6.1 ± 2.4, *p* = 0.01) that were observed in the active group compared with the placebo group. A borderline significant difference was apparent in the total eye symptom score (mean diff ± SE: −12.5 ± 7.5, *p* = 0.07) with the active group tending to improve more than the placebo group (Table 4b and Figure 3).

Rescue medication intake did not confound nasal and eye symptom scores in intention-to-treat and subgroup analyses.

### Quality of Life

Data availability for intention-to-treat analysis (*n* = 53) and subgroup analysis (*n* = 34) was similar to that for symptom analysis by TNESS (Q1), albeit with a slightly smaller sample for subgroup analysis, as 36% (19 out of 53) of participants had started data entry for the weekly Q2 later than peak 2 (Table 5).

**TABLE 5:** Quality of life.

	Variable	Group		Baseline	12 weeks	Within group	Active vs. placebo between groups	
					
			*N*	Mean ± *SD*	Mean ± *SD*	Mean change ± *SD*	Mean diff ± SE	*p*-value

(a) All (*n* = 53)	Does hay fever influence …							
	**Recreational activities/sports**	**Active**	29	2.2 ± 1.4	1.4 ± 1.4	−0.9 ± 1.6	−0.4 ± 0.5	ns
		**Placebo**	24	2.1 ± 1.5	1.6 ± 1.3	−0.5 ± 1.7		
	**Gardening**	**Active**	29	2.2 ± 1.6	1.3 ± 1.4	−0.9 ± 1.7	−0.3 ± 0.5	ns
		**Placebo**	24	2.0 ± 1 3	1.4 ± 1.3	−0.6 ± 1.6		
	**Sleep**	**Active**	29	2.2 ± 1.7	1.3 ± 1.2	−1.0 ± 1.3	−0.6 ± 0.4	ns
		**Placebo**	24	2.0 ± 1.6	1.6 ± 2.0	−0.3 ± 1.9		
	**Do you feel…**							
	**Tired**	**Active**	29	2.3 ± 1.7	1.3 ± 1.1	−1.1 ± 1.3	−0.6 ± 0.4	0.06
		**Placebo**	24	2.2 ± 2.1	1.7 ± 2.0	−0.5 ± 1.5		
	**Thirsty**	**Active**	29	1.5 ± 1.6	0.8 ± 1.1	–0.7 ± 1.2	−0.9 ± 0.4	0.06
		**Placebo**	24	1.1 ± 1.3	1.2 ± 1.8	0.2 ± 1.8		
	**Irritable**	**Active**	29	2.0 ± 1.8	0.8 ± 0.9	−1.3 ± 1.6	−0.8 ± 0.4	0.07
		**Placebo**	24	1.7 ± 1.6	1.2 ± 1.4	−0.5 ± 1.7		
	**Headache**	**Active**	29	1.6 ± 1.8	0.9 ± 1.4	−0.7 ± 1.7	−0.3 ± 0.4	ns
		**Placebo**	24	1.6 ± 1.9	1.2 ± 1.6	−0.4 ± 1.5		

	**Does hay fever influence …**							

**(b) Subgroup (*n* = 34)**	**Recreational activities/sports**	**Active**	19	2.2 ± 1.1	1.4 ± 1.2	−0.8 ± 1.2	−0.9 ± 0.4	0.05

		**Placebo**	15	1.5 ± 0.9	1.6 ± 1.3	0.1 ± 1.3		
	**Gardening**	**Active**	19	2.2 ± 1.4	1.3 ± 1.2	−0.9 ± 1.5	−0.9 ± 0.5	0.07
		**Placebo**	15	1.4 ± 0.7	1.3 ± 1.3	−0.1 ± 1.3		
	**Sleep**	**Active**	19	2.5 ± 1.7	1.3 ± 1.0	−1.3 ± 1.2	−1.6 ± 0.5	0.005
		**Placebo**	15	1.5 ± 1.1	1.9 ± 2.1	0.3 ± 1.9		
	**Do you feel…**							
	**Tired**	**Active**	19	2.7 ± 1.9	1.6 ± 1.2	−1.2 ± 1.4	−1.3 ± 0.4	0.004
		**Placebo**	15	1.9 ± 2.0	1.9 ± 2.1	0.1 ± 1.0		
	**Thirsty**	**Active**	19	1.8 ± 1.7	1.0 ± 1.2	−0.8 ± 1.2	–1.5 ± 0.5	0.007
		**Placebo**	15	1.0 ± 1.3	1.7 ± 2.1	0.7 ± 1.9		
	**Irritable**	**Active**	19	2.3 ± 1.9	0.9 ± 0.8	−0.5 ± 1.6	−1.5 ± 0.5	0.007
		**Placebo**	15	1.3 ± 1.3	1.3 ± 1.4	0 ± 1.4		
	**Headache**	**Active**	19	1.9 ± 1.8	1.0 ± 1.3	−1.0 ± 2.0	−1.1 ± 0.6	0.07
		**Placebo**	15	1.2 ± 1.6	1.3 ± 1.6	0.1 ± 1.3		

*This table summarizes the variables in the Quality of Life Questionnaire (Supplementary Appendix 1) not covered in the Total Nasal and Eye Symptom Questionnaire (Supplementary Appendix 2).*

*(a) All available data with baseline peak 1 (12 October 2020) and end of study peak 10 (25 December 2020) adjusted for intention-to-treat analysis.*

*(b) Subgroup of participants who received full 10–12 week intervention and provided baseline data before peak 3 (9 November 2020).*

*N, number; SD, standard deviation; ns, not significant.*

While the quality of life tended to improve in both groups, no significant differences were observed in the intention-to-treat analysis.

However, in the subgroup analysis of 34 participants who had taken the intervention for a 10–12 week period, a significant improvement in the active group compared with the placebo group was observed in functionality during the day (mean diff ± SE: −0.9 ± 0.45, *p* = 0.05), better sleep (mean diff ± SE: −1.6 ± 0.5, *p* = 0.005), less fatigue (mean diff ± SE: −1.3 ± 0.4, *p* = 0.04), less thirst (mean diff ± SE:-1.5 ± 0.5, *p* = 0.007), less irritability (mean diff ± SE: −1.5 ± 0.5, *p* = 0.007), and borderline significantly fewer headaches (mean diff ± SE: −1.1 ± 0.6, *p* = 0.07) (Table 5).

### Weekly Gastrointestinal Symptoms: Tolerability

Besides four participants withdrawing early in the trial due to gastrointestinal disturbances, the trial powder was generally well tolerated by the *n* = 60 participants.

Comparison of gastrointestinal symptoms between baseline (at enrollment) and end of study (end of December 2020) in the intention-to-treat analysis found the active group to report significantly lesser flatulence than the placebo group (in week 5: *p* = 0.01).

Other gastrointestinal symptoms such as bloating and burping tended to be less bothersome in the active group than in the placebo group, albeit not statistically significant.

**FIGURE 4:**
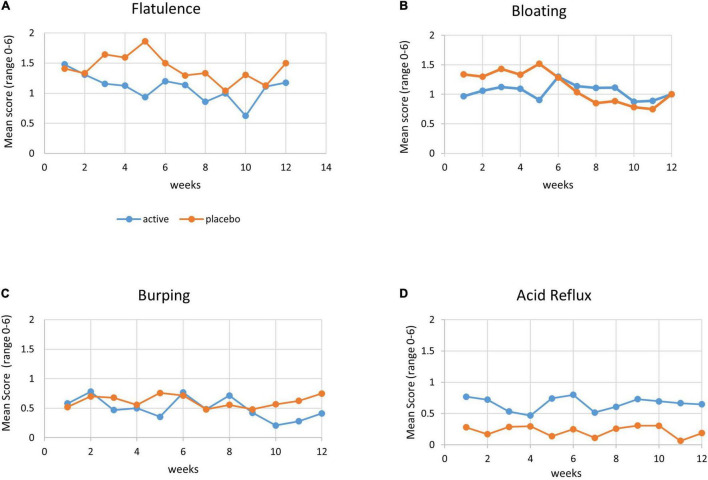
Gastrointestinal symptoms. Mean score of gastrointestinal symptoms (range 0–6, with 0 = no symptoms and 6 = severe symptoms) by week, with active group (blue line) and placebo group (orange line). **(A)** Flatulence, **(B)** bloating, **(C)** burping, and **(D)** acid reflux.

Acid reflux was significantly lower in the placebo group compared with the active group at all-time points including baseline, indicative of this symptom to be independent of the trial supplement (Figure 4).

### Cytokine Analysis

We compared cytokine levels between the groups at pollen peak 10 (25 December 2020) at the end of the study (*n* = 46). We did not have baseline cytokine levels before pollen exposure available as most participants enrolled during the pollen season. However, a comparison of cytokine levels at the end of the study allowed insight into inflammatory response to the allergen/pollen after the intervention.

Two groups of cytokines were analyzed, group one included the pro-inflammatory cytokines, which trigger a T-helper cell response known as Th1, and these were IL1, IL6, IL7, IL8, IL17, INFγ, TNFα, and TNFβ. Group 2 included the anti-inflammatory cytokines, which trigger a T-helper cell response known as Th2, and these were IL2, IL3, IL4, IL5, IL10, IL12, IL13, and GMSF.

Significant differences in cytokine levels between the groups were observed for pro-inflammatory cytokines IL6 (mean diff ± SE: −9.5 ± 3.3 pg/ml, *p* = 0.007), IL17 (mean diff ± SE: −1.6 ± 0.6 pg/ml, *p* = 0.01), INFγ (mean diff ± SE: −30.2 ± 14.4 pg/ml, *p* = 0.04), and TNFβ (mean diff ± SE: −28.8 ± 12.9 pg/ml, *p* = 0.03), with lower levels in the active group (Table 6).

**TABLE 6:** Cytokines (*N* = 46).

Variable	Group		Peak	Active vs. placebo between groups	
		
		*N*	Mean ± *SD* pg/ml	Mean diff ± *SE* pg/ml	*p*-value
**Pro-inflammatory Th1**					
**IL1**	**Active**	25	0.1 ± 0.1	−0.1 ± 0.1	ns
	**Placebo**	21	0.2 ± 0.4		
**IL6**	**Active**	25	3.6 ± 4.6	−9.5 ± 3.3	0.007
	**Placebo**	21	13.0 ± 15.9		
**IL7**	**Active**	25	1.5 ± 1.1	−0.4 ± 0.4	ns
	**Placebo**	21	1.8 ± 1.5		
**IL8**	**Active**	25	16.1 ± 18.0	4.3 ± 4.6	ns
	**Placebo**	21	11.8 ± 12.1		
**IL17**	**Active**	25	0.3 ± 0.2	−1.6 ± 0.6	0.01
	**Placebo**	21	2.0 ± 3.1		
**INF-γ**	**Active**	25	6.6 ± 6.7	−30.2 ± 14.4	0.04
	**Placebo**	21	36.8 ± 71.7		
**TNF-α**	**Active**	25	1.9 ± 1.6	−1.0 ± 0.7	ns
	**Placebo**	21	2.9 ± 3.0		
**TNF-β**	**Active**	25	6.3 ± 2.0	−28.8 ± 12.9	0.03
	**Placebo**	21	35.1 ± 65.6		
**Anti-inflammatory Th2**					
**IL2**	**Active**	25	2.0 ± 4.7	−2.4 ± 1.6	ns
	**Placebo**	21	4.3 ± 6.3		
**IL3**	**Active**	25	2.4 ± 0.9	−1.5 ± 1.1	ns
	**Placebo**	21	3.9 ± 5.3		
**IL4**	**Active**	25	1.0 ± 0.7	−2.3 ± 1.0	0.03
	**Placebo**	21	3.3 ± 5.1		
**IL5**	**Active**	25	3.6 ± 4.4	−6.2 ± 2.8	0.03
	**Placebo**	21	9.8 ± 13.0		
**IL10**	**Active**	25	0.3 ± 0.6	−0.6 ± 0.3	ns
	**Placebo**	21	0.9 ± 1.5		
**IL12**	**Active**	25	0.4 ± 0.3	−0.1 ± 0.1	ns
	**Placebo**	21	0.5 ± 0.4		
**IL13**	**Active**	25	0.4 ± 0.1	−0.8 ± 0.4	0.04
	**Placebo**	21	1.2 ± 2.1		
**GMSF**	**Active**	25	20.8 ± 6.7	−6.4 ± 4.2	ns
	**Placebo**	21	27.2 ± 19.9		

*N, number; SD, standard deviation; SE. standard error; ns, not significant; pg/ml, pictogram/ml; Th, T-helper cell; IL, interleukin; INF, interferon; TNF, tumor necrosis factor; GMSF, granulocyte colony-stimulating factor or macrophage colony-stimulating factor.*

In the anti-inflammatory cytokine group, significant differences between the groups were observed for IL4 (mean diff ± SE: −2.3 ± 1.0 pg/ml, *p* = 0.03), IL5 (mean diff ± SE: −6.2 ± 2.8 pg/ml, *p* = 0.03), and IL13 (mean diff ± SE: −0.8 ± 0.4 pg/ml, *p* = 0.04), with lower levels in the active group (Table 6).

As no direct measurement of T-helper cells was available, we used the formula “pro-inflammatory cytokine divided by anti-inflammatory cytokine” as a proxy for the Th1/Th2 ratio, an indicator of inflammatory profile. A lower Th1/Th2 ratio is an indicator of a lower inflammatory response. To ascertain meaningful Th1/Th2 ratios, we focused the analyses on those cytokines found to be significantly different between the groups.

Table 7 (Th1/Th2 ratio) summarizes those ratios, which were found to be significantly different between the groups. These include INFγ/IL4 (mean diff ± SE: −2.1 ± 0.9 pg/ml, *p* = 0.03), TNFβ/IL4 (mean diff ± *SE*: −2.0 ± 0.9 pg/ml, *p* = 0.02), and TNFβ/IL3 (mean diff ± *SE*: −11.4 ± 4.8 pg/ml, *p* = 0.02), with the active group showing a lower inflammatory profile than the placebo group.

**TABLE 7:** Th1/Th2 ratio (*N* = 46).

Variable	Group		Peak	Active vs. placebo between groups	
		
		*N*	Mean ± *SD* pg/ml	Mean diff ± SE pg/ml	*p*-value
**Th1/Th2 ratio**					
**INFγ /IL4**	**Active**	25	6.4 ± 1.6	−2.1 ± 0.9	0.03
	**Placebo**	21	8.5 ± 4.4		
**TNF**β**/IL4**	**Active**	25	7.1 ± 2.2	−2.0 ± 0.9	0.02
	**Placebo**	21	9.6 ± 4.5		
**TNF**β**/IL3**	**Active**	25	3.0 ± 1.2	−11.4 ± 4.8	0.02
	**Placebo**	21	8.1 ± 13.5		

*N, number; SD, standard deviation; SE. standard error; ns, not significant; pg/ml, pictogram/ml; Th, T-helper cell; IL, interleukin; INF, interferon; TNF, tumor necrosis factor; GMSF, granulocyte colony-stimulating factor or macrophage colony-stimulating factor.*

### Microbiome Analysis

**FIGURE 5:**
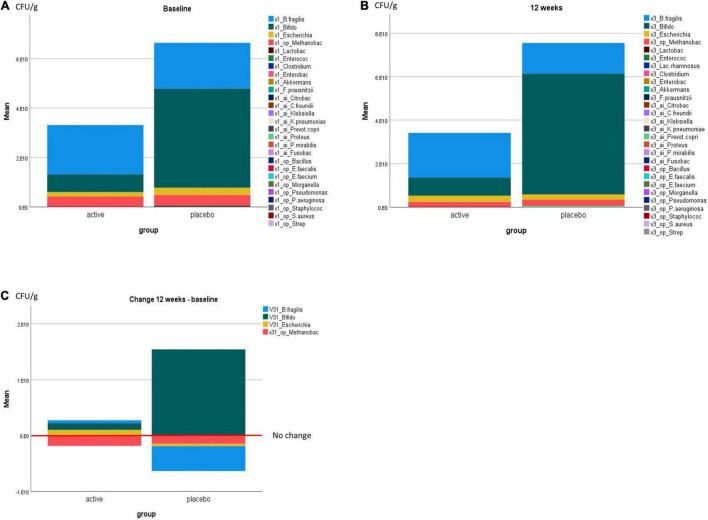
Microbial richness/number of bacteria in CFU/g by the trial group at **(A)** baseline, **(B)** 12 weeks, and **(C)** change at 12 weeks compared with baseline. All bacterial species tested for are listed in **(A,B)**, and only bacterial species with visible changes are displayed in **(C)**.

Four bacterial species groups changed visibly over the trial period, including the commensal bacteria *Bacteroides fragilis, Bifidobacteria, Escherichia coli*, and the opportunistic bacterial species of *Methanobacteriaceae*. Marked changes were not significantly different between the groups for three species, while a borderline significant increase in *E. coli* was observed in the active group compared with the placebo group (mean ± SE: 1.5 E10 ± 0.8 E10 CFU/g, *p* = 0.07) (Figure 5 and Table 8).

**TABLE 8:** Microbiome change at 12 weeks compared with baseline.

			Within group		Active vs. placebo between groups		
Bacteria species	Group	*N*	Mean change CFU/g	SD CFU/g	Mean diff CFU/g	*SE*	*P*-value
V31_*Bifidobacteria*	Active	28	1.17 E9	1.8 E10	−1.4 E10	1.2 E10	ns
	Placebo	29	1.54 E10	6.1 E10			
V31_*Bacteroides fragilis*	Active	28	5.4 E8	4.6 E10	5.0 E9	1.0 E9	ns
	Placebo	29	−4.44 E9	3.1 E10			
V31_*Enterococcus*	Active	28	4.9 E6	3.1 E7	−1.4 E7	1.4 E7	ns
	Placebo	29	1.9 E7	6.7 E7			
V31_*Escherichia*	Active	28	1.1 E9	3.1 E9	1.5 E9	8.3 E8	0.07*
	Placebo	29	−4.6 E8	3.2 E9			
V31_*Lactobacillus*	Active	28	2.8 E7	2.4 E8	5.3 E7	5.2 E2	ns
	placebo	29	−2.5 E7	1.4 E8			
V31_*Clostridia*	Active	28	−4.6 E6	2.6 E7	−6.4 E6	7.0 E6	ns
	Placebo	29	1.8 E6	2.7 E7			
V31_*Enterobacter*	Active	27	1.4 E7	1.5 E8	6.8 E6	2.8 E7	ns
	Placebo	29	6.9 E6	3.6 E7			
V31_*Akkermansia*	Active	16	5.7 E3	2.8 E4	3.3 E3	9.1 E3	ns
	Placebo	19	2.5 E3	2.6 E4			
V31_*F.prausnitzii*	Active	28	5.5 E4	5.1 E5	4.7 E4	1.3 E5	ns
	Placebo	29	7.9 E3	4.9 E5			
v31_ai_*Fusobacteria*	Active	27	−8.5 E6	3.4 E6	−8.7 E6	6.3 E6	ns
	Placebo	29	1.8 E5	4.4 E6			
v31_op_*Streptococcus*	Active	26	−1.9 E3	1.6 E5	571	4.1 E3	ns
	Placebo	25	−2.4 E3	1.3 E5			
v31_op_*Methanobacteriaceae*	Active	28	−1.8 E9	1.9 E9	−4.0 E8	5.7 E8	ns
	Placebo	27	−1.4 E9	2.3 E9	−4.0 E8	5.7 E8	
v31_*Bacteroidetes*	Active	28	2.1 E10	4.9 E11	−3.5 E12	3.5 E12	ns
	Placebo	29	3.6 E12	1.8 E13			
v31_*Firmicutes*	Active	28	−6.2 E10	2.9 E11	−5.3 E10	7.7 E10	ns
	Placebo	29	−9.4 E9	2.9 E11			
v31_FBratio	Active	28	−0.0350	0.18695	0.01707	0.05048	ns
	Placebo	29	−0.0521	0.19390			

*CFU/g, colony-forming units per gram stool; E10, 10^10^; v31, change between end from baseline; ai, potential autoimmune triggers; op, opportunistic bacteria; FB ratio, Firmicutes-to-Bacteroidetes ratio.*

Interestingly, the placebo group started with a visibly higher *Bifidobacteria* content, which further increased over the trial period. While *Bifidobacteria* were part of the active trial powder, the placebo trial powder contained fructooligosaccharides or prebiotics, which may have contributed to the observed changes. Notably, the commensal bacteria *Bacteroides fragilis* increased in the active group, while it decreased in the placebo group. In both groups, the opportunistic *Methanobacteriaceae* decreased (Figure 5).

Changes in clinically relevant *Clostridia* and *Bacteroides* species were minor and not significantly different between the groups (Table 8).

Several bacterial species were measurable only in a small proportion of participants (*n* < 10 in each group), and therefore, generalizability of the observed changes is limited. These included the autoimmune triggering *Citrobacter freundii* (active/placebo: *n* = 6/2), *Klebsiella pneumonia*, and opportunistic bacteria: *Bacillus* species, *Enterococcus faecalis*, *Enterococcus faecium*, *Staphylococcus* species, and *Staphylococcus aureus* **(data not shown).**

No significant differences were observed in the change of *Bacteroidetes* and *Firmicute*s species and the *Bacteroidetes* to *Firmicutes* ratio (Table 8).

### Blinding

Blinding was successful, with the majority (88%) in the probiotic group and 79% in the placebo group being unsure or incorrect in guessing their allocated group (Table 9).

**TABLE 9:** Blinding.

	Probiotic (*n* = 31)		Placebo (*n* = 29)		*p*-value
	*N*	%	*N*	%	
Correct	4	13	6	21	ns
Incorrect	7	23	5	17	ns
Unsure	20	65	18	62	ns

*ns, not significant.*

## Discussion

Our study suggests the NC Seasonal Biotic probiotic formula, if taken for a 10–12 week period during the hay fever season, to be effective in reducing symptoms, such as runny nose (*p* = 0.04) and itchy eyes (*p* = 0.01). Consequently, probiotic intake improved participants’ quality of life, including better functionality during the day (*p* = 0.05), better sleep (*p* = 0.005), less fatigue (*p* = 0.04), less thirst (*p* = 0.007), and less irritability (*p* = 0.007) compared with placebo.

Intention-to-treat analysis of all participants was hampered by the slow recruitment due to COVID-19-related restrictions and lengthy lockdowns in Victoria, Australia during the October–December 2020 hay fever period. Therefore, one-third of the participants were not in a position to take the trial supplement for the trial period of 3 months and rather took it for only 4–8 weeks.

This non-optimal shortened intervention period likely influenced the effectiveness of the treatment and confounded outcome measures in intention-to-treat analysis; however, clear trends of greater improvements in symptoms and quality of life were observed in the active group compared with the placebo group.

Our results are in line with the literature, whereby probiotic intake has been associated with improvements in symptoms and quality of life in sufferers with seasonal allergy (2, 3).

Furthermore, immunological parameters assessed by pro- and anti-inflammatory cytokines as a proxy for the T-helper cell Th1/Th2 ratio during the pollen peak at the end of this study improved significantly in the probiotic group compared with the placebo group, in line with the literature (2, 9, 10).

After the early withdrawal of four participants in the active group (10%) due to reported gastrointestinal intolerances, the probiotic formula was well tolerated, with no significant differences between the groups, except for significantly less flatulence in the active group in week 5 of the intervention.

Comprehensive microbial stool analysis revealed no significant changes in microbial richness or diversity in both groups. However, a marked increase in *Bifidobacteria* in the placebo group and a borderline significant increase of *E. coli* in the active group were noted.

This observed inverted change in *Bifidobacteria* count, while unexpectedly, may be explainable by the high prebiotic content in the form of fructooligosaccharides or prebiotics in the placebo trial powder furthering the growth of the already markedly higher *Bifidobacteria* content in the placebo group at baseline.

*Lactobacillus* bacteria, also provided in the active trial powder, did not appreciably change in either group.

The observed slight non-significant increase in *E. coli* bacteria in the active group is likely not clinically relevant. *E. coli* belongs to the commensal bacteria, which provide the host with essential nutrients. They metabolize indigestible compounds, defend against colonization of opportunistic pathogens, and contribute to the development of the intestinal architecture as well as stimulation of the immune system (18).

There are some limitations to this study, first, the smaller than planned sample size due to recruitment difficulties during COVID-19 restrictions and the high withdrawal rate, requiring us to continue recruitment throughout the hay fever season. Despite these obstacles, however, the subgroup analysis of 66% of participants who had taken the full 10–12 week course of trial intervention was adequately powered to reveal significant differences in symptoms and quality of life between the groups.

To improve recruitment for a study dependent on season, future trials may seek expressions of interest earlier. Earlier recruitment would also allow the collection of blood samples for cytokine analysis at baseline before pollen peak, allowing to assess individual changes in cytokine levels. In this study, we used cytokine analysis as a proxy for T-helper cell profiles similar to Kawase et al. (10) as direct analysis of T-cell by flow cytometry was not accessible, limiting direct comparability to those studies (9).

Furthermore, the placebo powder should ideally contain an inert substance, however, in this study, the placebo consisted mainly of prebiotics, which may influence the growth of bacteria in the microbiome. It is likely that the prebiotics contributed to the marked increase in Bifidobacteria in the placebo group, one of the species provided in the active probiotic powder.

## Conclusion

Our study suggests the NC Seasonal Biotic probiotic formula, if taken for 10–12 weeks, to be effective in reducing hay fever symptoms, such as runny nose and itchy eyes, and consequently improving the quality of life for sufferers with hay fever, with better functionality during the day, better sleep, less tiredness, and less irritability. The probiotic formula was well tolerated, and improved immunological parameters significantly in the probiotic group.

Future studies should begin recruitment well in advance to the start of the hay fever season to allow baseline assessment before pollen peaks and timely start of all participants at the beginning of the hay fever season.

## Data Availability Statement

The raw data supporting the conclusions of this article will be made available by the authors, without undue reservation.

## Ethics Statement

The studies involving human participants were reviewed and approved by the National Institute of Integrative Medicine Human Research Ethics Committee. The patients/participants provided their written informed consent to participate in this study.

## Author Contributions

KR and AS conceptualized the study. KR acquired funding. NT and YP oversaw the data collection. KR undertook the data analysis and prepared the manuscript with contributions from coauthors. All authors approved the final version.

## Conflict of Interest

The authors declare that the research was conducted in the absence of any commercial or financial relationships that could be construed as a potential conflict of interest.

## Publisher’s Note

All claims expressed in this article are solely those of the authors and do not necessarily represent those of their affiliated organizations, or those of the publisher, the editors and the reviewers. Any product that may be evaluated in this article, or claim that may be made by its manufacturer, is not guaranteed or endorsed by the publisher.

## Abbreviations

CFU/g: colony-forming units per gram stool
E10: 10^10^
v1: baseline
v3: 12 weeks
ai: potential autoimmune triggers
op: opportunistic bacteria.
