# Supplementary material for: Effects of a Probiotic Formulation on Seasonal Allergic Rhinitis in Adults—A Randomized Double-Blind Placebo-Controlled Trial: The Probiotics for Hay Fever Trial
Source: Front Nutr. 2022 May 23;9:887978. doi: 10.3389/fnut.2022.887978 (PMC9169690; doi:10.3389/fnut.2022.887978)
Supplement: Supplementary file 1 [file Data_Sheet_1.PDF]

Appendix Q1: Modified Mini Rhinoconjunctivitis Quality of Life Questionnaire (Mini RQLQ) Juniper 2000

On a scale from 0-6 with "0=not troubled" to "6=extremely troubled", how troubled have you been during the last week (month) as a result of your hayfever/ seasonal allergic rhinitis.

**Questions**	**Not troubled**	**Hardly at all troubled**	**Somewhat troubled**	**Moderately troubled**	**Quite a bit troubled**	**Very troubled**	**Extremely troubled**
Activities<br>**1. Regular activities at home and at work** (your occupation or tasks that you have to do regularly around your home and/or garden)	0	1	2	3	4	5	6
**2. Recreational activities** (indoor and outdoor activities with friend and family, sports, social activities, hobbies)	0	1	2	3	4	5	6
**3. Sleep** (difficulties getting a good night's sleep and/or getting to sleep at night)	0	1	2	3	4	5	6
3b. Snoring	0	1	2	3	4	5	6
**Practical problems**<br>4. Need to rub nose/eyes	0	1	2	3	4	5	6
5. Need to blow nose repeatedly	0	1	2	3	4	5	6
**Nose symptoms**<br>6. Sneezing	0	1	2	3	4	5	6
7. Stuffy blocked nose	0	1	2	3	4	5	6
8. Runny nose<br>incl. Post-nasal drip	0	1	2	3	4	5	6
9. Itchy nose or throat	0	1	2	3	4	5	6
**Eye symptoms**<br>10. Itchy eyes	0	1	2	3	4	5	6
11. Sore eyes	0	1	2	3	4	5	6
12. Watery eyes	0	1	2	3	4	5	6
13. Swelling/ puffy eyes (facial tenderness)	0	1	2	3	4	5	6
**Other symptoms**<br>14. Tiredness/ fatigue	0	1	2	3	4	5	6
15. Thirst	0	1	2	3	4	5	6
16. Feeling irritable	0	1	2	3	4	5	6
17. itchy skin/ ears	0	1	2	3	4	5	6
18. Headache<br>(severity & duration)	0	1	2	3	4	5	6
18b. duration – more details							

Screening: Reflect on how troubled you have been in last year's hayfever season
