# Supplementary material for: Effects of a Probiotic Formulation on Seasonal Allergic Rhinitis in Adults—A Randomized Double-Blind Placebo-Controlled Trial: The Probiotics for Hay Fever Trial
Source: Front Nutr. 2022 May 23;9:887978. doi: 10.3389/fnut.2022.887978 (PMC9169690; doi:10.3389/fnut.2022.887978)
Supplement: Supplementary file 2 [file Data_Sheet_2.PDF]

Appendix Q2: Total Nasal Symptom Score & Eye Symptom Score [Juniper 2000, Downie 2004, Wasserfallen 1997, Pfaar 2014]

On a scale from 0-6 with "0=not troublesome" to "6=extremely troublesome",
how troublesome have you found you are the following symptoms interfering with your normal daily activities or sleep?

SYMPTOMS	No symptoms	Mild symptoms		Moderate Symptoms		Severe Symptoms	
**How troublesome have you found you're the following symptoms interfering with your normal daily activities or sleep**	**Not troublesome**	**Hardly at all troublesome**	**Somewhat troublesome**	**Moderately troublesome**	**Quite a bit troublesome**	**Very troublesome**	**Extremely troublesome**
**NASAL**							
1. Stuffy blocked nose (nasal obstruction)	0	1	2	3	4	5	6
2. Itching/sneezing	0	1	2	3	4	5	6
3. Runny Nose Incl Post-nasal drip	0	1	2	3	4	5	6
**EYES**							
4. Ocular itching	0	1	2	3	4	5	6
5. Ocular grittiness (feeling like sand in the eye)	0	1	2	3	4	5	6
6. Ocular redness (redness in the eye)	0	1	2	3	4	5	6
7. Ocular tearing (watery eyes)	0	1	2	3	4	5	6
8. Ocular swelling/ puffy eyes (facial tenderness)	0	1	2	3	4	5	6
