# Supplementary material for: Effects of a Probiotic Formulation on Seasonal Allergic Rhinitis in Adults—A Randomized Double-Blind Placebo-Controlled Trial: The Probiotics for Hay Fever Trial
Source: Front Nutr. 2022 May 23;9:887978. doi: 10.3389/fnut.2022.887978 (PMC9169690; doi:10.3389/fnut.2022.887978)
Supplement: Supplementary file 3 [file Data_Sheet_3.PDF]

Appendix Q3: Gastrointestinal Symptom Rating Scale (GSRS) Revicki 1998

On a scale from 0-6 with “0=no discomfort al all” to “6=very severe discomfort”, how discomfortable have you been

Questions<br><br>**During the last week, have you been bothered by**	No discomfort at all	Slight discomfort	Mild discomfort	Moderate discomfort	Moderately severe discomfort	Severe discomfort	Very severe discomfort
**1. Stomach ache or pain**	0	1	2	3	4	5	6
**2. Heart burn** (pain behind breastbone in your chest)	0	1	2	3	4	5	6
**3. Acid reflux** (regurgitation or flow of sour or bitter fluid into your mouth)	0	1	2	3	4	5	6
4. **Hunger pains**	0	1	2	3	4	5	6
5. **Nausea**	0	1	2	3	4	5	6
6. **Rumbling** in your stomach	0	1	2	3	4	5	6
7. **Bloating**	0	1	2	3	4	5	6
8. **Burping**	0	1	2	3	4	5	6
9. **Passing gas/flatulence**	0	1	2	3	4	5	6
10. **Constipation / hard stools**	0	1	2	3	4	5	6
11. **Diarrhea / loose stools**	0	1	2	3	4	5	6
12. **urgent need to rush to the toilet**	0	1	2	3	4	5	6
13. **feeling of not completely emptying the bowel**	0	1	2	3	4	5	6
